# Comparison of three PCR-based assays for SNP genotyping in plants

**DOI:** 10.1186/s13007-018-0295-6

**Published:** 2018-03-28

**Authors:** Chiara Broccanello, Claudia Chiodi, Andrew Funk, J. Mitchell McGrath, Lee Panella, Piergiorgio Stevanato

**Affiliations:** 1 DAFNAE, Università degli Studi di Padova, Legnaro, Italy; 2 USDA-ARS Sugarbeet and Bean Research, East Lansing, MI USA; 3 USDA-ARS Sugarbeet Research, Fort Collins, CO USA

**Keywords:** Single nucleotide polymorphisms, Allelic discrimination assays, TaqMan, KASP, rhAmp

## Abstract

**Background:**

PCR allelic discrimination technologies have broad applications in the detection of single nucleotide polymorphisms (SNPs) in genetics and genomics. The use of fluorescence-tagged probes is the leading method for targeted SNP detection, but assay costs and error rates could be improved to increase genotyping efficiency. A new assay, rhAmp, based on RNase H2-dependent PCR (rhPCR) combined with a universal reporter system attempts to reduce error rates from primer/primer and primer/probe dimers while lowering costs compared to existing technologies. Before rhAmp can be widely adopted, more experimentation is required to validate its effectiveness versus established methods.

**Results:**

The aim of this study was to compare the accuracy, sensitivity and costs of TaqMan, KASP, and rhAmp SNP genotyping methods in sugar beet (*Beta vulgaris* L.). For each approach, assays were designed to genotype 33 SNPs in a set of 96 sugar beet individuals obtained from 12 parental lines. The assay sensitivity was tested using a series of dilutions from 100 to 0.1 ng per PCR reaction. PCR was carried out on the QuantStudio 12K Flex Real-Time PCR System (Thermo Fisher Scientific, USA). The call-rate, defined as the percentage of genotype calls relative to the possible number of calls, was 97.0, 97.6, and 98.1% for TaqMan, KASP, and rhAmp, respectively. For rhAmp SNP, 24 of the 33 SNPs demonstrated 100% concordance with other two technologies. The genotype concordance with either technologies for the other 9 targets was above 99% (99.34–99.89%).

**Conclusion:**

The sensitivity test demonstrated that TaqMan and rhAmp were able to successfully determine SNP genotypes using as little as 0.2 ng DNA per reaction, while the KASP was unable to ascertain SNP states below 0.9 ng of DNA per reaction. Comparative cost per reaction was also analyzed with rhAmp SNP offering the lowest cost per reaction. In conclusion, rhAmp produced more calls than either TaqMan or KASP, higher signal to NTC data while offering the lowest cost per reaction.

**Electronic supplementary material:**

The online version of this article (10.1186/s13007-018-0295-6) contains supplementary material, which is available to authorized users.

## Background

Plant breeding in the twenty first century uses the three pillars of phenotypic data, genetic variation, and molecular markers to develop improved crop varieties for a growing world [1]. Molecular marker technology is constantly improving, increasing genotypic resolution while reducing time and costs [2]. Molecular markers are a useful tool for monitoring the presence of key qualitative and quantitative traits based on a few large-effect loci, especially when the costs of phenotyping greatly exceed the costs of genotyping. Correctly understanding the trade-offs between different genotyping approaches is important for plant breeders to make the best decisions to maximize efficient crop improvement.

Single-nucleotide polymorphisms (SNPs) are currently the most widely-used molecular markers due to their ubiquitous distribution throughout a given genome, as well as their low cost compared to other marker technologies [3, 4]. These markers are applicable across the full breadth of living organisms, providing universal interest in SNP technology development. Next-generation sequencing (NGS) technologies can detect large numbers of SNPs in breeding populations [5, 6]. A rise in the number of available SNP markers has led to increased demand for SNP genotyping capabilities, resulting in numerous cost-effective genotyping platforms available to researchers and breeders.

One of the earliest methods for SNP genotyping was the TaqMan system (Applied Biosystems, Foster City, CA) [7] based on fluorescently-tagged, allele-specific probes detected using real-time polymerase chain reaction (PCR)-based assays [8]. Another leading SNP genotyping technology is Kompetitive allele specific PCR (KASP) [6], which uses endpoint fluorescence detection to discriminate tagged alleles [9]. The TaqMan and the KASP assays have been widely used for genotyping due to their high-throughput, low cost, sensitivity and tolerance of variation in the quality and quantity of input DNA. In addition, the TaqMan and KASP assays are suitable for use on a variety of real-time PCR instruments [10]. However, these platforms still suffer from imperfect sensitivity and allele discrimination, along with less expensive costs of implementation.

A new method called rhAmp based on RNase H2-dependent PCR (rhPCR) has been developed in an effort to address weaknesses of TaqMan and KASP technologies. This method uses RNase H2 to activate primers after successful binding to their target sites, reducing primer dimer formation and improving the specificity of the reaction [11]. Because of the recent introduction of the rhAmp technology, the assay still needs validation to better understand how it performs compared to established methods such as TaqMan and KASP.

In this report, we compare the accuracy, sensitivity, cost and data analysis time of rhAmp-based SNP genotyping system with two existing methods, TaqMan and KASP. These three chemistries were tested with 33 SNP markers on 12 inbred populations of *Beta vulgaris* (sugar beet). The assays were conducted using the high-throughput genotyping pipeline based on the QuantStudio 12K Flex real-time PCR machine (Life Technologies, CA, USA), allowing genotyping of 384 samples per run.

## Methods

### Plant Material and growing conditions

The study included 12 sugar beet genotypes from the DAFNAE-Department of Agronomy, Food, Natural resources, Animals and Environment (University of Padova, Italy) collection (Additional file 1: Table S1). This plant material was previously genotyped as part of a fingerprinting study [12]. All these accessions were diploid, multigerm, and homozygous for resistance to rhizomania (*Rz1*). Before planting, seeds were surface-disinfected by soaking in ethanol 96% for 5 min, rinsed with sterile water, then incubated overnight in 50 ml of 0.3% hydrogen peroxide to obtain a greater homogeneity in plant germination. Seeds were planted in plastic pots and grown in a peat-based potting mix. Plants were grown in a climatic chamber for one month. Pots were placed in a growth chamber set at day/night temperatures of 25/18 °C, relative humidity of 70/90%, a 14-h photoperiod, and an irradiance of 60 W m^−2^.

### DNA isolation and quantification

Eight plants representing eight biological replicates in each genotype were sampled individually. Approximately 0.2 g of tissue was taken for automated DNA isolation using the BioSprint 96 workstation (Qiagen, Hilden, Germany), following the procedure described by Stevanato et al. [13]. The quality and quantity of extracted DNA were evaluated by BioPhotometer (Eppendorf, Hamburg, Germany) and Qubit 2.0 Fluorometer (Thermo Fisher Scientific, Waltham, MA, USA), according to manufacturer’s suggestions. An analysis of DNA integrity was performed also by gel electrophoresis.

### SNP analysis

A set of pre-validated SNPs ([12]; Additional file 2: Table S2) was used to design TaqMan, KASP and rhAmp assays. Supplementary material contains the flanking sequences with evaluated SNPs indicated by brackets. All custom SNP assays were designed by the corresponding companies (Thermo Fisher, LGC Genomics and Integrated DNA Technology, respectively). TaqMan, KASP and rhAmp assays were performed in 5 μl using 384-well plates low Rox was used as a passive reference dye. The allelic specificity of TaqMan assay was provided by two probes, one labelled with FAM dye and the other with VIC dye. As regards KASP and rhAmp assays, bi-allelic discrimination was achieved through the competitive binding of two allele specific forward primers, one labelled with FAM dye and the other with HEX dye for KASP, one labelled with FAM dye and the other with Yakima Yellow (YY) dye for rhAmp. These different reporter dyes were detected independently on real-time qPCR instruments with excitation sources and emission filters in the respective wavelengths.

The three reaction mixes are reported in Table 1.

**Table 1:** Composition of reaction mixes required per sample in a total volume of 5 μl

	Master Mix (μl)	Assay Mix (μl)	H_2_O (μl)	DNA (μl)
TaqMan	2.5	0.125	1.375	1
KASP	2.5	0.07	–	2.43
rhAmp	2.65	0.25	1	1.1

- *TaqMan genotyping assay* Genotyping by TaqMan was performed following manufacture’s instruction using 10 ng of DNA mixed with the TaqMan Genotyping Master Mix (Catalogue number: 437135, Thermo Fisher Scientific) and custom TaqMan SNP assays (Thermo Fisher Scientific).
- *KASP genotyping assay* Genotyping by KASP was performed following manufacture’s instruction using 10 ng of DNA mixed with the KASP Genotyping Master Mix (Catalogue number: KBS-1016-017, LGC Genomics, Hoddesdon, UK) and custom KASP SNP assays (LGC Genomics).
- *rhAmp genotyping assay* Genotyping by rhAmp was performed following manufacture’s instruction using 5 ng of DNA mixed with rhAmp Genotyping Mix, composed by rhAmp Genotyping Master Mix (catalogue number: 1076017, Integrated DNA Technology, Coralville, Iowa, USA), rhAmp Reporter Mix (catalogue number: 1076028, Integrated DNA Technology) and custom rhAmp SNP assays (Integrated DNA Technology).

PCR assays were prepared using a QIAgility liquid-handling robot (Qiagen). The robot was also set to pipette 96 individual DNAs per each run. The reactions were run in duplicates and with 4 non-template controls (NTC) in each run and for each custom assay. The total number of analyses in each assay was: 33 SNPs × 12 genotype × 8 plants in each line × 2 technical replication = 6392 analyses. Plates were sealed with adhesive film (MicroAmp Optical Adhesive Film, Thermo Fisher Scientific) and briefly centrifuged (5000*g*, 30 s) before thermal cycling. Thermal cycling parameters are described in Table 2. Allelic discrimination plots were created using the QuantStudio 12K Flex software for genotyping.

**Table 2:** Thermal cycling parameters for each of the three real-time PCR protocols

	Pre-read stage	Hold stage	1st PCR stage		2nd PCR stage		Post-read stage
	°C/Time	°C/Time	°C/Time	Cycles	°C/Time	Cycles	°C/Time
TaqMan	60 °C/30 s	95 °C/10 min	95 °C/15 s	40			60 °C/30 s
			60 °C/1 min				
KASP	30 °C/1 min	94 °C/15 min	94 °C/20 s	10	94 °C/20 s	29	30 °C/1 min
			61–55 °C/1 min (dropping 0.6 °C per cycle)		55 °C/1 min		
rhAmp	60 °C/30 s	95 °C/10 min	95 °C/10 s	37			60 °C/30 s
			60 °C/30 s				
			68 °C/20 s				

### Evaluation of quantitative metrics

**Fig. 1:**
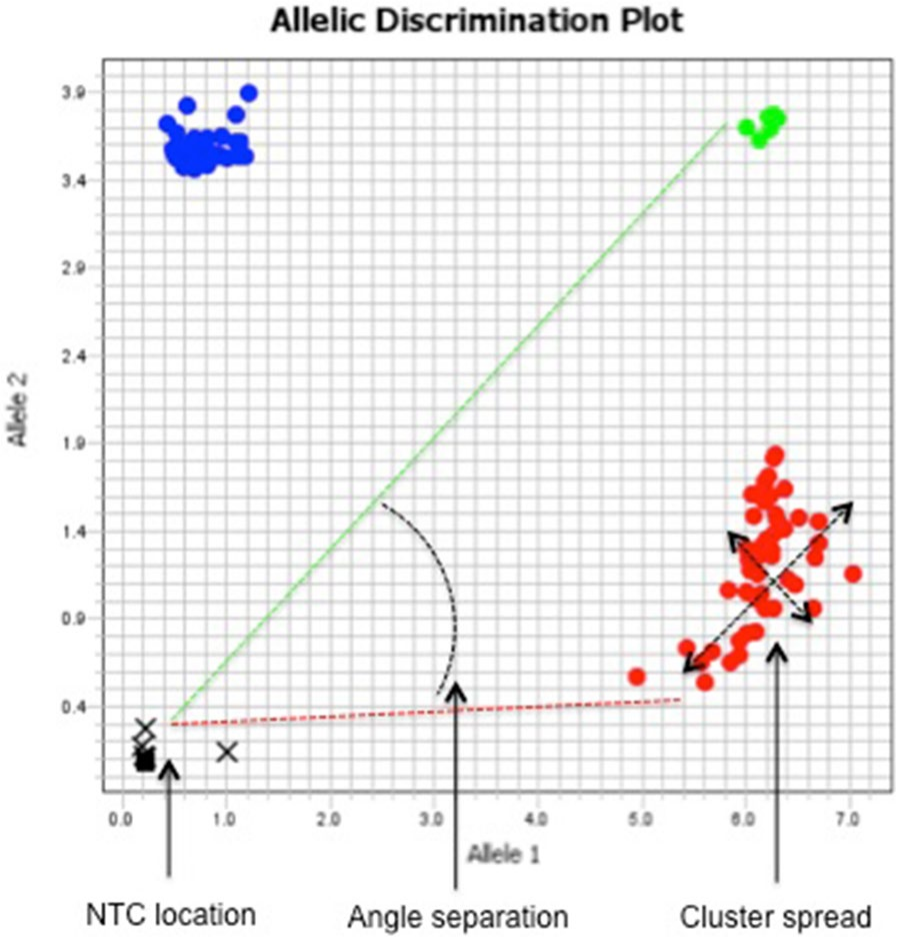
Schematic representation of quantitative metrics calculated using the FAM and VIC Rn values from instrument software

Quantitative metrics such as “Call rate”, “No template control (NTC) location”, “Cluster to NTC distance”, “Cluster angle separation” and “Cluster spread” were calculated using the FAM and VIC Rn values from instrument software. Figure 1 shows a schematic representation of the evaluated metrics.

- Call rate is the percentage of successful genotype calls per passing SNP.
- No template control (NTC) location is the mean of FAM Rn and VIC Rn value of NTC reactions.
  $$Mean\;FAM\;Rn_{NTC} = \frac{{\sum FAM\;Rn_{NTC} }}{n}$$
  $${\text{Mean}}\;{\text{VIC}}\;{\text{Rn}}_{\text{NTC}} = \frac{{\sum {\text{VIC}}\;{\text{Rn}}_{\text{NTC}} }}{n}$$
  where n = number of no template control reactions.
- Cluster to NTC distance is the mean distance of all data points in each cluster to NTC.
  $$Cluster\;to\;NTC\;distance = \frac{{\sum \sqrt {\left( {FAM\;Rn_{i} - Mean\;FAM\;Rn_{NTC} } \right)^{2} + (VIC\;Rn_{i} - Mean\;VIC\;Rn_{NTC} )^{2} } }}{n}$$
  where n = number of data points in each cluster.
- Cluster angle separation is the angle difference between Homozygous allele clusters and Heterozygous cluster. Cluster angle is calculated as the mean angle of all data points in each cluster with respect to the x-axis and NTC cluster as the origin.
  $$Cluster\;angle\;separation = \frac{{\sum \tan^{-1} \left( {\frac{{\left( {VIC\;Rn_{i} - Mean\;VIC\;Rn_{NTC} } \right)}}{{(FAM\;Rn_{i} - Mean\;FAM\;Rn_{NTC} )}}} \right)}}{n}$$
  where n = number of data points in each cluster.
- Cluster spread is the standard deviation of distance between each data points to the center of cluster.
  $$Cluster\;spread = \sqrt {\frac{{\sum \left( {D - \bar{D}} \right)^{2} }}{n}}$$
  where ${\text{D}} = \sqrt {\left( {FAM\;Rn_{i} - \frac{{\sum FAM\;Rn_{i} }}{n}} \right)^{2} + \left( {VIC\;Rn_{i} - \frac{{\sum VIC\;Rn_{i} }}{n}} \right)^{2} }$, $\bar{D} = \frac{\sum D}{n}$, n = number of data points in each cluster.

### Sensitivity test

An analytical sensitivity test was performed at various DNA input ranging from 100 to 0.1 ng DNA per reaction. The limit of detection (LOD) of each method was evaluated using Rn, commonly referred as normalized reporter value.

### Data analysis

The data were subjected to analysis of variance using Statistica 10.0 package (StatSoft Inc., Tulsa, OK, USA) and Fisher’s Protected Least Significant Difference was calculated for mean comparison.

## Results

**Fig. 2:**
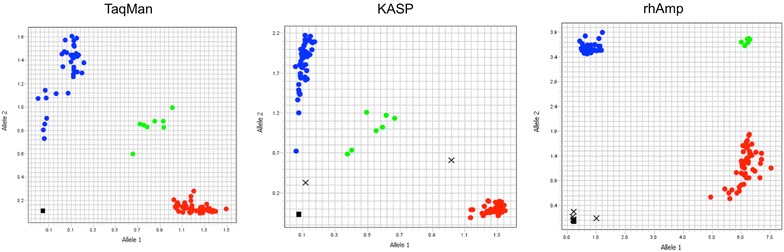
Allelic discrimination plots obtained for SNP103 using TaqMan, KASP and rhAmp genotyping assays on 96 samples. Red and blue dots represent the homozygous genotypes, the green circles represent heterozygous genotypes and the squares on the bottom left of the plot are no-template control

TaqMan, KASP and rhAmp assays successfully genotyped 29 out of the 33 SNPs on the 12 genotypes. SNP21, SNP204, SNP262 and SNP176 showed high failure rates across all three approaches and were withheld from subsequent analysis. Figure 2 shows an example of clustering among sugar beet individuals obtained with SNP103 TaqMan, KASP and rhAmp assays.

Table 3 shows ANOVA analysis for the differences among TaqMan, KASP and rhAmp assays in quantitative metrics. The rhAmp assay gave a higher call rate than those of TaqMan and KASP even if they were not significantly different. Highly significant differences (P < 0.001) were found among the three assays for cluster to NTC distance and Cluster spread metrics. The rhAmp assay showed higher cluster to NTC distance and lower Cluster spread with respect to TaqMan and KASP, respectively (Table 4).

**Table 3:** Analysis of variance (ANOVA) showing the effect of TaqMan, KASP and rhAmp assays on quantitative metrics

Trait	Effect	df	Sum of squares	Mean squares	*F*	*P*
Call rate	Assay	2	0.031	0.016	2.5	0.090
	Error	96	0.612	0.006		
	Total	98	0.643			
Cluster to NTC distance	Assay	2	178.135	89.067	185.6	< 0.001
	Error	96	46.078	0.480		
	Total	98	224.213			
Cluster angle separation	Assay	2	82.305	41.152	0.5	0.605
	Error	96	7813.594	81.392		
	Total	98	7895.898			
Cluster spread	Assay	2	0.683	0.342	38.8	< 0.001
	Error	96	0.844	0.009		
	Total	98	1.528

**Table 4:** Average values for analysis of variance (ANOVA) showing the effect of TaqMan, KASP and rhAmp assays on quantitative metrics

Assay	Call rate	No template control (NTC) location	Cluster to NTC distance	Cluster angle separation	Cluster spread
TaqMan	0.970a	0.85a	2.09b	43.96a	0.18b
KASP	0.976a	0.14b	1.72c	42.95a	0.16b
rhAmp	0.981a	0.86a	4.73a	41.73a	0.10a

Different letters means significant difference at P < 0.05 according to Fisher’s Protected Least Significant Difference. No template control (NTC) location is the mean of four NTC reactions

We analyzed the concordance rate of the three different methods, excluding any SNP with missing data in one or more samples. This resulted in exclusion of 1233 calls due to missing data. Thus, the concordance between the three chemistries was calculated on the 87% of theoretical maximum data, representing 2757 SNP/genotype combinations, each called with TaqMan, KASP and rhAmp. There were 32 cases of discordance between assays (1.16%), comprised of disagreement concerning heterozygous versus homozygous SNP state. We found that the concordance rates between KASP and TaqMan, and between KASP and rhAmp were 99.24 and 98.94%, respectively.

The limit of detection (LOD) for SNP genotyping was determined for each method using a dilution series of input gDNA ranging from 100 to 0.1 ng DNA per reaction. Results showed that TaqMan and rhAmp were able to successfully genotype SNPs with as little as 0.2 ng DNA per reaction, while the KASP was unable to genotype SNPs at gDNA input lower than 0.9 ng per reaction (Table 5). All three assays failed to call SNPs at 0.1 ng per reaction.

**Table 5:** Normalized fluorescence levels (ΔRn values) obtained for SNP103 from the 20-fold dilutions using the three chemistries

DNA input (ng)	TaqMan		KASP		rhAmp	
	Allele1	Allele2	Allele1	Allele2	Allele1	Allele2
100	1.5	2.3	0.7	1.1	5.9	3.4
90	1.4	2.2	0.6	0.5	5.9	3.5
80	1.6	2.2	0.6	0.6	5.9	3.6
70	1.6	2.3	0.6	0.6	5.9	3.6
60	1.6	2.4	0.6	0.5	6.1	3.7
50	1.6	2.4	0.7	0.6	6.1	3.6
40	1.6	2.3	0.7	0.6	6.1	3.6
30	1.5	2.3	0.7	0.8	5.9	3.4
20	1.4	2.2	0.8	0.6	5.3	2.5
10	1.4	2.3	0.6	0.6	1.6	1.9
1	0.6	1.7	0.4	0.3	2.8	1.4
0.9	0.9	1.4	0.3	0.4	1.5	1.6
0.8	0.9	1.7	0.2	0.2	2.3	0.9
0.7	0.9	1.6	0.2	0.3	1.5	0.7
0.6	0.7	1.4	0.2	0.3	1.0	1.1
0.5	0.4	1.6	0.2	0.1	1.1	1.2
0.4	0.8	0.9	0.1	0.2	1.5	0.9
0.3	0.5	1.4	0.1	0.1	1.6	0.9
0.2	0.9	0.7	0.1	0.1	0.8	1.0
0.1	0.1	0.8	0.1	0.1	0.1	0.1
Water	0.0	0.1	0.1	0.2	0.1	0.1

Each chemistry provides two ΔRn values, one for each allele. Samples above the level of detection (italics text) were genotyped. Samples below the level of detection (regular text) resulted undetermined

A cost estimate for three SNP genotyping methods is shown in Table 6. It is based on the PCR reaction volume at 5 μl in a 384-well plate. The assay cost includes PCR primers, fluorescent probes and master mix. The cost of rhAmp assays is lowest at 0.10 €/sample, while TaqMan assay cost was highest at 0.29 €/sample. It should be noted that the cost of Master Mix was estimated based on 10 or 25 ml size.

**Table 6:** Cost per sample for each chemistry

	Cost (€)	No. samples	€/sample	Tot €/sample
TaqMan				
Assay	233.12	1500	0.16	
MasterMix	534.8 (10 ml)	4000	0.13	0.29
KASP				
Assay	45.87	5000	0.01	
MasterMix	987.53 (25 ml)	10,000	0.10	0.11
rhAmp				
Assay	54	1500	0.03	
MasterMix	741.9 (25 ml)	10,000	0.07	0.10

The cost was obtained considering the cost of the assay mix (primers and probes) and the cost of the master mix

Time for 384-wells plate preparation and data analysis is reported in Table 7. In general, all three methods are similar in total plate set-up and PCR run time ranging from 125 min from TaqMan, 115 min for KASP and 110 min for rhAmp.

**Table 7:** Time required for 384-wells plate preparation and run time for each chemistry

	TaqMan	KASP	rhAmp
Plate preparation (min, 384 wells-plate)	30	30	30
Run time (min)	95	85	80
Total (min)	125	115	110

Plates were prepared using a QIAgility liquid-handling robot (Qiagen)

## Discussion

Determining the genetic composition of organisms and tissues is critical to diverse scientific pursuits. Improvements in the accuracy, time per run and cost of genotyping technologies have led to their widespread use in fields such as medicine, food safety, basic research and plant breeding. In the dynamic environment of molecular biology, it is challenging to keep up with the evolution of new technologies and methods. Here, we provide a comparison of a new genotyping assay, rhAmp, with two established methods, TaqMan and KASP. We focus the attention on quantitative metrics such as call rate, sensitivity, concordance, and the way in which these values differ across the three chemistries. For each method, we compare also the genotyping cost per sample and data turnaround time.

The three PCR allelic discrimination technologies proved to be reliable allowing high-throughput genotyping. All the approaches present distinct and clear clusters in the allelic discrimination plots. The call rate was consistently above 97% with the highest values reported for rhAmp. In addition, rhAmp and TaqMan showed the highest concordance rate, above 99%. Discordant calls and undetermined results between the three chemistries may be due in part to variations in quantity and quality of DNA that can affect particularly TaqMan and KASP.

As a result, rhAmp provides higher sensitivity, together with TaqMan, and more distinct clusters in the allelic discrimination plot. The sensitivity of each assay was tested to determine the lower limit of DNA required for successful allelic discrimination and defined as the lowest amount of target DNA detected by the assay [14]. It has also been evaluated that distance between no-template control and clusters, which is directly proportional to the fluorescence level and greater for rhAmp with respect to that of TaqMan and KASP.

SNP genotyping calls can be determined by end-point PCR or combination of end-point and real-time PCR. TaqMan and rhAMP can perform both end-point and real-time PCR while KASP is end-point only [6, 15]. The advantage of assays with both real-time and end-point data is to allow not only automatic SNP genotyping calls but also manual calls of undetermined samples by further evaluating the fluorescence signal over PCR cycles [16]. It is particularly useful for assays with higher non-specific signals between alleles 1 and 2 which often result in SNP genotyping clusters collapsed at the very late PCR stage.

In all three evaluated techniques, allelic discrimination depends on hybridization in which a single base varies, and in certain cases, non-specific hybridization can occur [2]. rhAMP technology use blocked primers to minimize primer-dimer formation and non-specific amplification [11]. Coupling cleavage by RNase H2 to primer extension increases the specificity of the assays. This particular feature of rhAmp led the assay to be used in multiplex PCR. On the other hand, KASP and TaqMan assays can be used only in singleplex.

Looking at the cost per data point, based on our estimation, TaqMan is the most expensive method, while KASP and rhAmp provided much greater flexibility in terms of cost. Both rhAmp and KASP did not require labelling of the SNP-specific primers, which reduce the cost compared to TaqMan. Yuan et al. [5] report the same issue, quantifying the cost of KASP and TaqMan for the soybean genotyping as 0.005 US$ and 0.238 US$ respectively. In addiction, genotyping cost is determined by the size of the PCR reaction volume [2]. Considering that the three chemistries required flexible SNP platform, they can be run in a variety of real-time PCR instruments. This allows the use of the assays also in 1536 well plates. TaqMan technology can be optimized also in the OpenArray platform, where the assays are pre-loaded into OpenArray plates, providing up to 3072 reactions at 33 nl volumes.

In all the three tests we observed low genotyping error rates, low labor cost, and tolerance of variations in quantity and quality of DNA. These properties are critical for plant selection in breeding programs because the optimization for a large number of DNA samples prior to conduct PCR reaction is not easily applicable.

The three chemistries had largely similar concordance in SNP calling, with the rhAmp chemistry providing marginal increases in accuracy over KASP, as well as significant cost reductions compared with TaqMan. We conclude that rhAmp provides an improvement in the efficiency of SNP detection compared to TaqMan and KASP. These results should assist plant breeders in making informed decisions on how to use their resources to maximize gains through molecular breeding.

## Additional files

**Additional file 1: Table S1.** Description of the sugar beet genotypes used in this study.

**Additional file 2: Table S2.** Flanking sequences of evaluated SNPs indicated by brackets.

## Abbreviations

NGS: next generation sequencing
MAS: marker assisted selection
SNP: single nucleotide polymorphism
NTC: no template control
